# α-Hydroxybutyrate Is an Early Biomarker of Insulin Resistance and Glucose Intolerance in a Nondiabetic Population

**DOI:** 10.1371/journal.pone.0010883

**Published:** 2010-05-28

**Authors:** Walter E. Gall, Kirk Beebe, Kay A. Lawton, Klaus-Peter Adam, Matthew W. Mitchell, Pamela J. Nakhle, John A. Ryals, Michael V. Milburn, Monica Nannipieri, Stefania Camastra, Andrea Natali, Ele Ferrannini, for the the RISC Study Group

**Affiliations:** 1 Metabolon, Inc., Research Triangle Park, North Carolina, United States of America; 2 RISC (Relationship of Insulin Sensitivity to Cardiovascular Disease) Coordinating Office, Department of Internal Medicine, University of Pisa School of Medicine, Pisa, Italy; University of Tor Vergata, Italy

## Abstract

**Background:**

Insulin resistance is a risk factor for type 2 diabetes and cardiovascular disease progression. Current diagnostic tests, such as glycemic indicators, have limitations in the early detection of insulin resistant individuals. We searched for novel biomarkers identifying these at-risk subjects.

**Methods:**

Using mass spectrometry, non-targeted biochemical profiling was conducted in a cohort of 399 nondiabetic subjects representing a broad spectrum of insulin sensitivity and glucose tolerance (based on the hyperinsulinemic euglycemic clamp and oral glucose tolerance testing, respectively).

**Results:**

Random forest statistical analysis selected α-hydroxybutyrate (α–HB) as the top-ranked biochemical for separating insulin resistant (lower third of the clamp-derived M_FFM_ = 33 [12] µmol·min^−1^·kg_FFM_^−1^, median [interquartile range], n = 140) from insulin sensitive subjects (M_FFM_ = 66 [23] µmol·min^−1^·kg_FFM_^−1^) with a 76% accuracy. By targeted isotope dilution assay, plasma α–HB concentrations were reciprocally related to M_FFM_; and by partition analysis, an α–HB value of 5 µg/ml was found to best separate insulin resistant from insulin sensitive subjects. α–HB also separated subjects with normal glucose tolerance from those with impaired fasting glycemia or impaired glucose tolerance independently of, and in an additive fashion to, insulin resistance. These associations were also independent of sex, age and BMI. Other metabolites from this global analysis that significantly correlated to insulin sensitivity included certain organic acid, amino acid, lysophospholipid, acylcarnitine and fatty acid species. Several metabolites are intermediates related to α-HB metabolism and biosynthesis.

**Conclusions:**

α–hydroxybutyrate is an early marker for both insulin resistance and impaired glucose regulation. The underlying biochemical mechanisms may involve increased lipid oxidation and oxidative stress.

## Introduction

Insulin resistance (IR) has been established as a precursor of type 2 diabetes (T2D) [1], [2], [3], [4], [5], [6] and cardiovascular disease [7], [8], [9], [10], [11]. IR and compensatory hyperinsulinemia are commonly found in a variety of conditions, including obesity. When coupled with β-cell dysfunction, IR is a major pathophysiological determinant of dysglycemia (impaired fasting glycemia, IFG, and impaired glucose tolerance, IGT) and T2D [12], [13]. Conditions of high cardiovascular (CVD) risk such as hypertension, dyslipidemia, and atherosclerosis have also been associated with IR [12], [13], [14], [15]. However, our current understanding of these associations is incomplete.

Traditional clinical tests do not measure IR directly and, as a result, a variety of methods have been developed: the gold standard hyperinsulinemic euglycemic clamp (HI clamp); insulin tolerance test; steady state plasma glucose (SSPG) following fixed somatostatin/glucose/insulin infusions; and modeling analysis of the oral glucose tolerance test (OGTT) or frequently sampled intravenous glucose tolerance test (FSIVGTT) [16]. However, such procedures are mostly confined to clinical research settings due to cost and time constraints. Fasting insulin and derived indices (HOMA, QUICKI) have been widely used [17], but lack of insulin measurement standardization strongly limits their accuracy and has prevented adoption in routine clinical practice. The identification of novel markers for detection of IR subjects remains an unmet need. Further, this approach may reveal markers that are useful for identifying individuals at risk of progression to T2D and CVD, whereby enabling implementation of effective strategies for disease prevention and patient monitoring.

The RISC study (Relationship of Insulin Sensitivity to Cardiovascular Risk), comprising a nondiabetic cohort, was initiated to address how IR may contribute to T2D and CVD progression. We report here on a global biochemical profiling technology developed for the discovery of new biochemical biomarkers. This technology has been successfully applied to identify biochemicals associated with disease, toxicity and aging [18], [19], [20]. Here it was applied to identify biochemicals associated with IR and dysglycemia in 399 subjects, a subset of the RISC cohort, in which insulin sensitivity was measured directly by the HI clamp. We found that α-hydroxybutyrate (α–HB) is the most significant metabolite associated with insulin sensitivity and, interestingly, as an early marker for dysglycemia. The biochemical pathway for α–HB and its potential involvement in IR and dysglycemia are briefly discussed. Monitoring changes in the concentration of α–HB in fasting human plasma may provide novel insights on how early stages of IR evolve into T2D or CVD.

## Results

### Biochemical Profiling Analysis

**Figure 1:**
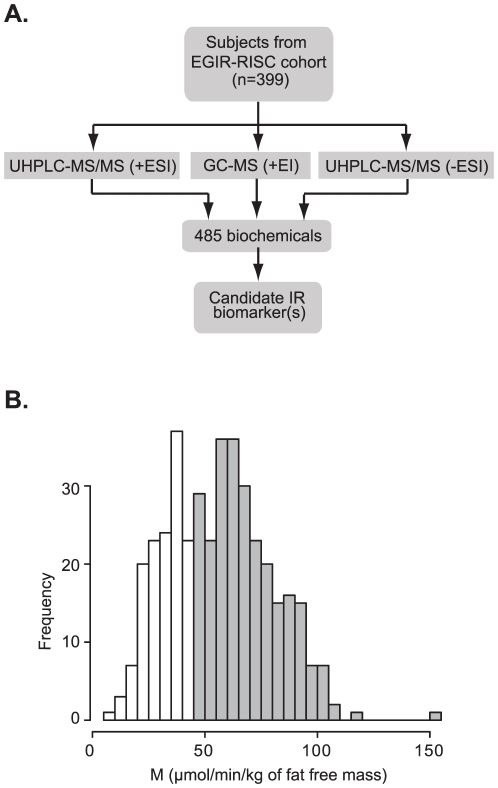
Global biochemical profiling analysis of a nondiabetic population. A. Metabolomic analysis schema. Plasma samples collected from 399 fasting nondiabetic subjects were analyzed on three separate mass spectrometry platforms. Ultra-high pressure liquid chromatography-mass spectrometry (UHPLC-MS) was performed in positive (+ESI) and negative (-ESI) ionization mode and gas chromatography (GC-MS) in positive ionization mode (+EI). An average of ∼485 biochemicals was measured in each sample. B. The distribution of insulin-mediated glucose disposal rates, expressed as M_FFM_ values (µmol·min^−1^·kg_FFM_^−1^), of 399 subjects selected from the RISC cohort and comprised of NGT, IGT, and IFG subjects. IR was defined as M≤45 µmol·min^−1^·kg_FFM_^−1^ as measured by clamp, representing the bottom third of the entire RISC cohort (n = 1293). Shaded bars insulin sensitive (IS); open bars insulin resistant (IR).

Fasting plasma samples from the RISC cohort were analyzed in a non-targeted fashion on three separate mass spectrometry platforms, UHPLC-MS/MS (+/- ESI) and GC-MS (+EI), with 485 biochemicals measured, as illustrated in **Figure 1A**. Each participant's insulin sensitivity was measured using the hyperinsulinemic euglycemic (HI) clamp; the distribution of M_FFM_ (M_FFM_ = insulin-mediated glucose disposal rate, µmol·min^−1^·kg_FFM_^−1^) in the 399 RISC subjects analyzed is shown in **Figure 1B**. Taking a commonly used classification approach [11], [21], [22], [23], the bottom tertile of insulin sensitivity of the entire EGIR-RISC cohort (n = 1293) (*i.e.*, M_FFM_≤45 µmol·min^−1^·kg_FFM_^−1^) was defined as IR. By this criterion, M_FFM_ was 33 [12] µmol·min^−1^·kg_FFM_^−1^, median [interquartile range], in the IR group (n = 140) and 66 [23] µmol·min^−1^·kg_FFM_^−1^ in the more insulin sensitive (IS) subjects. The demographic and metabolic characteristics of the 399 subjects under analysis are described in **Table 1**.

**Table 1:** Demographic and clinical characteristics of study subjects*.

Group	N	Parameter	Gender	Age (years)	BMI (kg/m^2^)	2-hour glucose (mg/dl)	M_FFM_
NGT-IS	211	Mean	121 f	44	24.1	91	71
		Median	90 m	43	23.7	90	68
NGT-IR	45	Mean	20 f	44	25.8	99	35
		Median	25 m	45	26.2	99	37
IGT	82	Mean	45 f	46	26.2	156	36
		Median	37 m	45	26.1	151	32
IFG	61	Mean	19 f	48	29.3	110	50
		Median	42 m	49	28.8	110	45

*M_FFM_ is expressed in µmol·min^−1^·kg_FFM_^−1^; 2-hour plasma glucose levels from the OGTT.

### α–HB is inversely associated with insulin sensitivity

**Figure 2:**
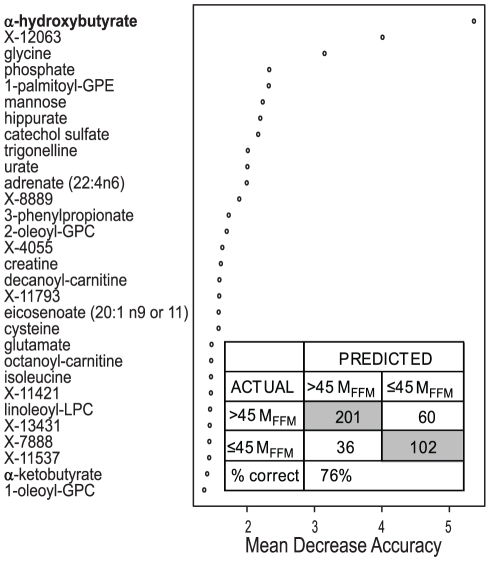
Classification of subjects as insulin sensitive or insulin resistant. Random Forest statistical analysis of biochemical profiling (metabolomic) data. The Importance Plot rank of metabolites according to the contribution of each to the classification of 399 subjects into insulin sensitive (IS, M_FFM_>45 µmol·min^−1^·kg_FFM_^−1^, top two-thirds of subjects, n = 261) or insulin resistant (IR, M_FFM_<45 µmol·min^−1^·kg_FFM_^−1^, bottom third of subjects, n = 138) groups. Metabolites are listed on the y-axis in order of importance, decreasing in importance from the top to bottom. The mean decrease in accuracy for each metabolite is plotted on x-axis. INSET: The Confusion Matrix showing the prediction accuracy of the separation of the top two-thirds (IS) from the bottom third (IR) is ∼76%.

To assess the ability to classify subjects as IS or IR, Random Forest (RF) analysis was performed. As shown in **Figure 2**, the organic acid, α–hydroxybutyrate (α–HB) was the top-ranked metabolite in the resulting importance plot, which ranks the classifiers based upon contribution of each to the separation of the subjects into classes. In this analysis the subjects were classified as either IS or IR with approximately 76% accuracy (inset). This result did not change when normalizing the M value for kg of body weight rather than kg of fat-free mass (data not shown).

Univariate correlation analysis of the data from the biochemical profiling screen also ranked α–HB as the metabolite with the highest correlation to the glucose disposal rate (r = −0.45, *p*-value 1.40E-21, **Table 2**). α–HB negatively correlated with total glucose disposal for both M_FFM_ (fat free mass, µmol·min^−1^·kg_FFM_^−1^) and M_WBM_ (whole body mass, mg·min^−1^·kg^−1^, data not shown). Summarized in **Table 2** are additional candidate biomarkers correlative to insulin sensitivity as measured by the euglycemic clamp (M_FFM_) with overlap observed with the initial RF analysis (**Figure 2**).

**Table 2:** Correlation of IR-related metabolites with M_FFM_ based upon global biochemical screen results.*

Biochemical	Correlation (*r*) M_FFM_	*p*-value
α-hydroxybutyrate (α-HB)	−0.45	1.40E-21
X-12063	−0.36	7.92E-14
glycine	0.33	2.79E-11
urate	−0.31	3.90E-10
X-12816	0.30	1.24E-09
α-ketobutyrate (α-KB)	−0.28	1.54E-08
catechol-sulfate	0.27	6.16E-08
trigonelline (N-methylnicotinate)	0.26	7.86E-08
phosphate	0.24	8.18E-07
decanoylcarnitine	0.24	8.89E-07
X-11440	−0.24	1.88E-06
3-methyl-2-oxovalerate	−0.23	3.12E-06
3-methyl-2-oxobutyrate	−0.23	3.24E-06
mannose	−0.23	4.45E-06
octanoylcarnitine	0.23	5.11E-06
adrenate (22:4n6)	−0.22	7.35E-06
cysteine	−0.22	7.65E-06
creatine	−0.22	9.05E-06
glycerate	0.22	1.07E-05
caprylate (8:0)	0.22	1.20E-05
quinate	0.22	1.48E-05
1-palmitoylglycerophosphoethanolamine	0.21	2.55E-05
isoleucine	−0.21	2.81E-05
isovalerylcarnitine	−0.21	3.13E-05
X-12844	−0.21	3.32E-05
myo-inositol	0.20	3.70E-05
X-11421	0.20	3.91E-05
X-4055	−0.20	5.02E-05
indolepropionate	0.20	6.29E-05
X-11537	0.19	9.33E-05

*Correlation coefficient with M_FFM_ of the 30 top-ranked metabolites identified by Random Forest are presented.

M_FFM_ is expressed in µmol·min^−1^·kg_FFM_^−1^.

**Figure 3:**
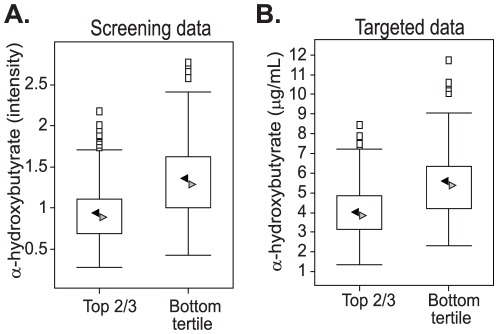
α-HB levels are higher in insulin resistant subjects in both screening and targeted assays. A. Box plot of α-HB levels measured in the non-targeted MS analysis (screening data). The X-axis shows the groups and the Y-axis shows the relative normalized intensity for α-HB median scaled to 1. B. Box plot of α-HB concentrations measured using targeted isotopic dilution assays (targeted data). The X-axis shows the groups and the Y-axis shows α-HB concentration in µg/ml. In the box plots the top and bottom of the box represent the 75th and 25th percentile, respectively. The top and bottom bars (“whiskers”) represent the entire spread of the data points for α-HB and each group, excluding “extreme” points, which are indicated with black squares. The black arrowheads indicate the mean value and the gray arrowheads indicate the median value.

Since the initial analyses were based upon relative quantification data obtained from the non-targeted biochemical profiling technology, a targeted assay was developed to provide absolute quantitative results. As shown in **Figure 3**, α–HB was consistently higher (*p*<0.0001 for both the screening and targeted data) in IR subjects compared to IS subjects, whether measured by the screening platform or by the targeted isotopic dilution assay.

### α–HB in dysglycemic subjects

**Figure 4:**
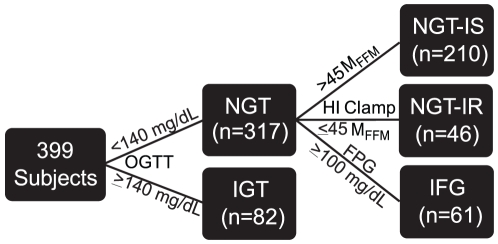
Classification of subjects according to insulin sensitivity and plasma glucose regulation. Schema showing the partitioning of the 399 subjects into groups according to the results of the oral glucose tolerance test (OGTT), fasting plasma glucose levels (FPG) and M values derived from the clamp. NGT, normal glucose tolerant; IGT, impaired glucose tolerant; NGT-IS, normal glucose tolerant and insulin sensitive (M_FFM_>45 µmol·min^−1^·kg_FFM_^−1^); NGT-IR, normal glucose tolerant and insulin resistant (M_FFM_≤45 µmol·min^−1^·kg_FFM_^−1^); IFG, normal glucose tolerant and impaired fasting glucose.

Subjects were classified as normoglycemic or dysglycemic based upon the results of fasting plasma glucose (FPG) and the oral glucose tolerance test (OGTT) as illustrated in **Figure 4**. Subjects with 2-hour glucose levels <7.8 mmol/l were deemed normal glucose tolerant (NGT) while those with 2-hour glucose between 7.8–11.1 mmol/l were deemed as having impaired glucose tolerance (IGT). Individuals with fasting plasma glucose levels ≥5.6 mmol/l were classified as having impaired fasting glucose (IFG). Thus, based on insulin sensitivity and glucose tolerance subjects were classified into four categories: NGT insulin sensitive (NGT-IS); NGT insulin resistant (NGT-IR); IFG; and IGT. Among NGT subjects, 46 (25 males, 21 females) were insulin resistant (NGT-IR), with an age of 45 [11], median [interquartile range], years and a BMI of 24.5 [4.7] kg·m^−2^. The 210 NGT subjects who were more insulin sensitive (NGT-IS) (90 males, 120 females) were 44 [13] years of age and had a BMI of 23.5 [4.2] kg·m^−2^. The 61 IFG subjects (42 males and 19 females) had an age of 49 [12] years and a BMI of 27.3 [4.8] kg·m^−2^, while the 82 IGT subjects (37 males and 45 females) had an age of 45 [13] years and a BMI of 27.9 [5.4] kg·m^−2^.

**Figure 5:**
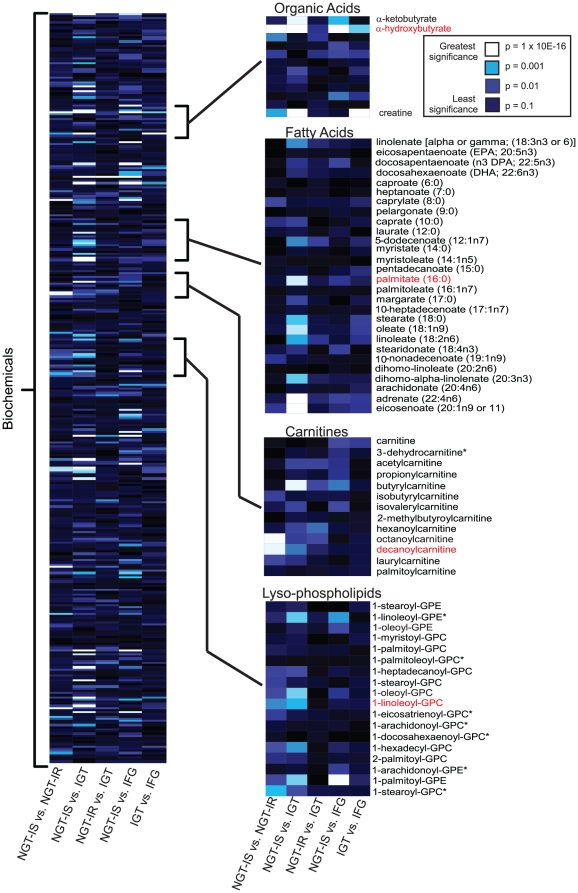
Biochemicals showing significant change in subjects with IR and/or dysglycemia. A heat map graphical representation of *p*-values obtained from statistical analysis of the global biochemical profiling of metabolites measured in plasma collected from NGT-IS, NGT-IR, IGT, and IFG subjects. *t*-tests were performed to determine those metabolites that significantly increase or decrease in insulin resistant (IR) and dysglycemic individuals (IGT, IFG). Highlighted from the main heat map include an organic acid, α-HB, the top-ranked biochemical for separating NGT-IS from NGT-IR and NGT-IS from IGT; a cluster of long-chain fatty acids such as palmitate that are pronounced when comparing NGT-IS to IGT; and acyl-carnitines and acylglycerophosphocholines that distinguish NGT-IR and IGT from NGT-IS. The color coding used, from white to dark blue, indicate the most significant to least significant, respectively, with white, most statistically significant (*p*≤1.0E-16); light blue (1.0E-16≤*p*≤0.001), royal blue (0.001≤*p*≤0.01), and dark blue, not significant (*p*≥0.1).

Shown in **Figure 5** is a heat map of the global biochemical profiling data set illustrating the statistical significance of changes in the biochemicals in the various pair-wise group comparisons. Four classes of metabolites that differentiate NGT-IS from NGT-IR and/or NGT-IS from dysglycemia (IFG or IGT) are highlighted. The organic acids α-ketobutyrate (α-KB), α-HB and creatine readily distinguish NGT-IS subjects from both IGT and IFG subjects, whereas α-HB and creatine serve as early indicators of IR by readily distinguishing NGT-IS from NGT-IR subjects. Similarly, lipid species such as acylcarnitines and lysoglycerophospholipids also distinguish NGT-IS and NGT-IR subjects and NGT-IS from IGT, with high statistical significance. In contrast, fatty acids such as palmitate are later stage markers of impaired glucose regulation, and only distinguish NGT-IS from IGT subjects in the continuum of insulin resistance.

### Targeted analysis of metabolites correlative of insulin sensitivity

**Figure 6:**
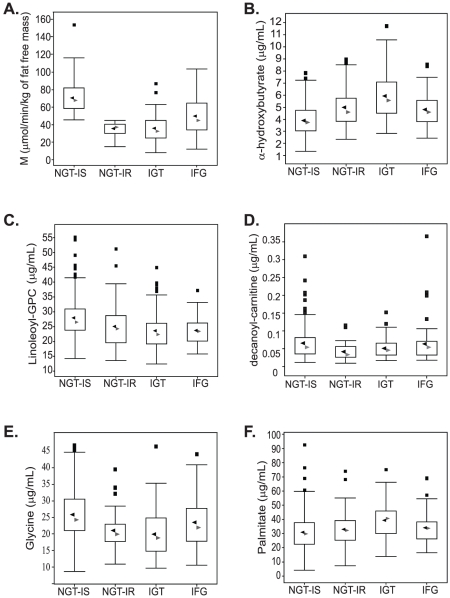
Insulin-mediatedglucose disposal rates and representative metabolite levels in insulin resistant and dysglycemic subjects. A. Box plots of insulin-mediated glucose disposal rates (M_FFM_, µmol·min^−1^·kg_FFM_^−1^); derived from the clamp in normoglycemic (NGT) and dysglycemic (IGT, FPG) subjects. B. – F. Box plots of concentrations (µg/ml) of representative metabolites that change significantly with insulin resistance and/or dysglycemia as measured by isotopic dilution assays in subjects with normal glucose tolerance that are insulin sensitive (NGT-IS) or insulin resistant (NGT-IS) and in dysglycemic subjects with impaired glucose tolerance (IGT) or impaired fasting plasma glucose levels (IFG). B. α-HB, C. linoleoyl-GPC, D. decanoyl-carnitine, E. glycine, F. palmitate.

Consistent with previous reports [24], M_FFM_ was significantly lower in each of the IFG, IGT, and NGT-IR groups in comparison with the NGT-IS group (p<0.0001 for each), as illustrated in **Figure 6A**, while plasma α–HB concentrations (**Figure 6B**), were the mirror image of M_FFM_. Using the targeted assay, the measured levels of α–HB were significantly (p<0.0001) higher in the NGT-IR, IFG and IGT groups as compared to the NGT-IS group. Relatedly, by partition analysis, an α–HB concentration of 5 µg/ml was found to best separate IR from IS subjects. Furthermore, based upon multiple logistic regression analysis, α–HB was significantly associated with IR independently of center (collection site), sex, age, and BMI, with an odds ratio of 2.84 (C.I.: 2.02–4.00, p<0.0001) for each SD ( = 1.7 µg/ml) of plasma α–HB.

Interestingly, RF analysis ranked α-HB as the most important metabolite to classify NGT and IGT subjects, with a >70% classification accuracy (data not shown). Consistent with these observations, α–HB levels were significantly higher in IGT than NGT subjects (p<0.0001), as shown in **Figure 6B**. To test whether α-HB levels segregated with glucose dysregulation in general, we grouped together IFG and IGT into one IGT category, and by multiple logistic analysis α-HB was significantly associated with IGT independently of center, sex, age, and BMI, with an odds ratio of 2.51 (C.I.: 1.81–3.49, p<0.0001) for each SD of plasma α–HB. Furthermore, both IR and IGT were each independently associated with an α–HB concentration in the top tertile of its plasma concentrations (i.e., 5.9 [1.7] µg/ml), with respective odds ratios of 3.26 (C.I.: 1.83–5.81, p<0.0001) and 2.72 (C.I.: 1.51–4.92, p = 0.0009) after adjustment for center, sex, age, and BMI.

In addition to measuring α-HB by absolute quantitation, targeted assays were also developed for candidate IR biomarkers identified by RF and correlation analyses, with examples of representative biochemical classes highlighted in **Figure 5**. The results of these targeted assays are presented in **Figure 6C–F**. For example, the lysophospholipid 1-linoleoylglycerophosphocholine (**Figure 6C**) and long-chain acylcarnitines such as decanoylcarnitine (**Figure 6D**) decrease in concentration with increasing insulin resistance and dysglycemia. Similarly, levels of the amino acid glycine were observed to trend downward with IR (**Figure 6E**). In contrast, similar to α-HB, the saturated fatty acid palmitate is inversely correlated with insulin sensitivity (**Figure 6F**). Related to this latter finding, a direct relationship between fasting plasma α–HB concentrations and the mean free fatty acid (FFA) level during the clamp (which averaged 30 [40] µmol/l) was observed; this association was highly statistically significant (*r* ^2^ = 0.25, *p*<0.0001) even after adjusting for center, sex, age, and BMI (data not shown).

Summarized in **Table 3** are representative targeted assay results for top-ranking IR candidate markers, with regard to their correlation to M_FFM_ value and their fold changes in concentration from the bottom tertile to the top two-thirds of insulin sensitivity (green: decreased fold-change; red: increased fold-change). Consistent with the screening data, α–HB is highly correlated to the glucose disposal rate (r = 0.45, *p*-value 1.15E-21).

**Table 3:** Correlation with M_FFM_ and fold-change with IR of IR-related metabolites based upon targeted assays.*

	Correlation coefficient (*r*)		Fold change
Biochemical	M_FFM_ µmol·min^−1^·kg_FFM_^−1^	*p*-value	Insulin sensitive/Insulin resistant (top 2/3/bottom tertile)
α-HB	−0.45	1.15 e-21	1.38 ↑
1-linoleoyl-GPC	0.33	4.44 e-19	0.77 ↓
glycine	0.32	2.64 e-11	0.85 ↓
3-methyl-2-oxobutyrate	−0.30	3.17 e-11	1.13 ↑
1-oleoyl-GPC	0.28	1.56 e-09	0.82 ↓
creatine	−0.26	1.29 e-07	1.30 ↑
decanoylcarnitine	−0.25	4.24 e-07	0.73 ↓
octanylcarnitine	−0.20	4.40 e-05	0.79 ↓
1-stearoyl-GPC	−0.20	5.36 e-05	0.89 ↓
adrenate (22:4n6)	−0.19	9.51 e-05	1.19 ↑
stearate	−0.18	0.000315	1.17 ↑
1-palmitoyl-GPC	−0.17	0.0008423	0.90 ↓
palmitate (16:0)	−0.16	0.0013302	1.17 ↑
margarate	−0.15	0.0023516	1.14 ↑

*The correlations with M_FFM_ for the top 14 metabolites ranked by Random Forest are presented. Upward arrow (↑) indicates metabolite concentration increased in insulin resistant subjects; Downward arrow (↓) indicates metabolite concentration decreased in insulin resistant subjects.

## Discussion

Using a non-targeted biochemical screening approach in a large and well characterized cohort of nondiabetic subjects representing a wide spectrum of insulin sensitivity, we identified α–hydroxybutyrate (α–HB) as a biomarker segregating with clamp-derived IR in subjects with normal glucose tolerance. Furthermore, α–HB segregated with dysglycemia (IFG+IGT) independently of, and in addition to, IR. Importantly, these associations were independent of sex, age, and BMI. Thus, together with other biomarkers, α–HB may provide a diagnostic tool to identify IR and/or IGT earlier than currently used clinical tests.

**Figure 7:**
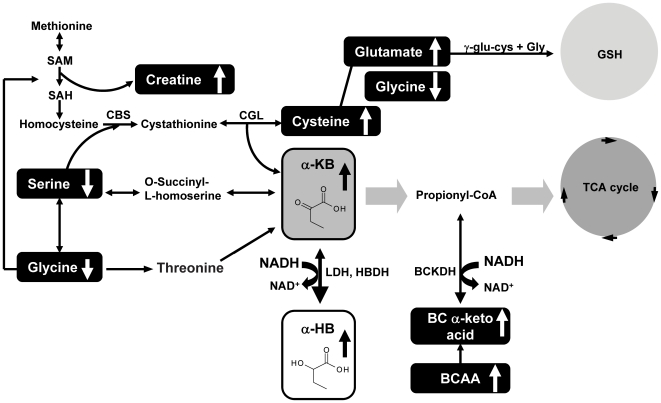
A Model of the biochemical relationship of α-HB biosynthesis and associated metabolic pathways with Insulin Resistance. α-HB is produced from the conversion of α-KB in a reaction catalyzed by LDH that occurs when the NADH/NAD^+^ ratio is elevated, as can occur from higher lipid oxidation events. Metabolites that change significantly (screening and targeted data, p<0.01) are indicated by a box; arrows indicate the direction of change. α-HB, alpha-hydroxybutyrate; α-KB, alpha-ketobutyrate; BCAA, branched chain amino acids; BCKDH, branched chain alpha keto acid dehydrogenase; CBS, cystathionine-beta-synthase; CGL, cystathionine gamma-lyase; HBDH, α-hydroxybutyrate dehydrogenase; LDH, lactate dehydrogenase; SAH, S-adenosyl-L-homocysteine; SAM, S-adenosyl Methionine. All compounds in boxes were measured using targeted assays, with the exception of α-KB, cysteine and BCAAs.

α–HB is an organic acid derived from α-ketobutyrate (α–KB) (**Figure 7**). α–KB is produced by amino acid catabolism (threonine and methionine) and glutathione anabolism (cysteine formation pathway) and is metabolized to propionyl-CoA and carbon dioxide [25]. α–HB is formed as a by-product during the formation of α–KB *via* a reaction catalyzed by lactate dehydrogenase (LDH) or α–hydroxybutyrate dehydrogenase (α–HBDH) (**Figure 7**), an LDH isoform present in the heart [26]. Accumulation of α–HB is postulated to occur *in vivo* when either (a) the formation of α–KB exceeds the rate of its catabolism, which leads to substrate accumulation, or (b) there is product inhibition of the dehydrogenase that catalyzes the conversion of α–KB to propionyl-CoA [25], [27].

α–KB is also produced as a result of the conversion of cystathionine to cysteine. Under conditions of increased oxidative stress, a higher flux of cysteine into production of glutathione, the primary antioxidant in cells, occurs from a shift in homocysteine production from transmethylation of methionine to transsulfuration of homocysteine to produce cystathionine [28] (**Figure 7**). In one report, α–HB was associated with excess glutathione demand and disrupted mitochondrial energy metabolism and shown to derive from hepatic glutathione stress [28], supporting the idea that elevated α–HB may be associated with increased oxidative stress in the IR state.

α–HB may become elevated by at least two mechanisms: (1) elevation of hepatic glutathione stress resulting in an increased demand for glutathione production, and (2) elevation of the NADH/NAD^+^ ratio due to increased lipid oxidation. The first mechanism likely contributes to increased α–HB formation by supplying more α–KB substrate from increased cysteine anabolism (**Figure 5**). Consistent with this interpretation, we observe statistically significant elevation of both α-KB and cysteine with increasing insulin resistance from the global screening data (**Figures 2** **&** **5** **,** **Table 2**), similar to the trend observed with α-HB. In support of the second proposed mechanism, increased lipid oxidation is a metabolic feature of IR, and is indexed by the insulin-inhibited FFA concentration [7], [14]. Our finding of a positive association between steady state FFA and plasma α–HB concentrations in the whole cohort supports the possibility that an increased NADH/NAD^+^ ratio favors reduction of α–KB to α–HB (**Figure 7**).

Changes in other important IR-associated metabolites within metabolic pathways leading to the formation of α-KB and α-HB are highlighted in **Figure 7**. For example, reduced levels of glycine (**Figure 6E**) and serine upstream of α-KB formation may be consistent with increased gluconeogenesis which is observed with IR in db-/db- mice [29]. Our interpretation that a redox imbalance may contribute to elevated α-HB in the context of IR is consistent with our finding that branched-chain alpha-keto acids, such as 3-methyl-2-oxobutyrate, are elevated with IR (**Table 3**). These increases may be due to the effect of the redox imbalance on the directionality of the dehydrogenases that reduce/oxidize these keto acids (**Figure 7**). In addition, α-HB has also been observed to be elevated in T2D subjects and animal models of T2D, as well as in severe lactic acidosis and ketoacidosis [25], [27], [30], [31], [32], [33], [34]. Interestingly, in normal subjects and T2D patients, it has been shown that restoration of the NADH/NAD^+^ redox balance by glutathione infusion therapy resulted in improvement of insulin sensitivity and β-cell function in normal subjects and in T2D patients [35].

In a recent study comparing the urinary profiles of 98 intermediary metabolites measured by targeted MS in 74 obese and 67 lean individuals, Newgard *et al.* identified a metabolic signature for the accumulation of branched-chain amino acids, the glutamine/glutamate couple, several acylcarnitines, and some aromatic amino acids (phenylalanine and tyrosine) using principal component analysis [36]. These metabolites were also related to insulin resistance (as determined by the HOMA index) and interpreted as marking the metabolic consequences of excessive fat and protein intake, with impairment of insulin signaling and mitochondrial overload. It is noteworthy that in the non-targeted metabolomics approach of the present study, lipid molecules, branched-chain amino acids, and acylcarnitines were also featured among the top 30 metabolites that RF analysis associated with the M value (**Figure 2**). The current data narrow down the complex interactions of amino acid and lipid metabolism [37] to highlight the importance of a single marker, α–HB, which may reflect oxidative burden in the context of IR.

With an unmet need for a practical clinical test that accurately measures IR in individuals, identification of α–HB as a significant biomarker for separating IR from IS subjects using a fasting plasma sample could lead to development of such a diagnostic test. α–HB in combination with other biochemical and clinical parameters may also prove to be useful as a clinical indicator of subclinical abnormalities of glucose metabolism.

## Methods

### Study subjects

RISC is a prospective, observational cohort study whose rationale and methodology have been published previously [38]. In brief, participants were recruited at 19 centers in 13 countries in Europe, according to following inclusion criteria: either sex, age 30–60 years, clinically healthy, stratified by sex and by age according to 10-year age groups. Initial exclusion criteria were: treatment for obesity, hypertension, lipid disorders or diabetes, pregnancy, cardiovascular or chronic lung disease, weight change of ≥5 kg in last month, cancer (in last 5 years), and renal failure. Exclusion criteria after screening were: arterial blood pressure ≥140/90 mmHg, fasting plasma glucose >7.0 mmol/l, 2-hour plasma glucose (on a standard 75-g oral glucose tolerance test [OGTT]) ≥11.0 mmol/l, total serum cholesterol ≥7.8 mmol/l, serum triglycerides ≥4.6 mmol/l, and ECG abnormalities. Baseline examinations began in June 2002 and were completed in July 2005.

Of 1293 clamped RISC subjects, 194 males and 205 females – median age 45 years and median body mass index (BMI) 25.0 kg m^−2^ (range 16.9–42.9) - were selected for non-targeted biochemical profiling analysis. Based on the OGTT, 256 subjects had normal glucose tolerance (NGT, *i.e.*, fasting plasma glucose <5.6 mmol/l and 2-hour glucose <7.8 mmol/l), 82 subjects had impaired glucose tolerance (IGT, *i.e.*, 2-hour glucose between 7.8–11.1 mmol/l), and 61 subjects had impaired fasting glycemia (IFG, *i.e.*, fasting glucose between 5.6–7.0 mmol/l).

EGIR-RISC study had undergone appropriate review by the European Commission research program and its ethics committee. Written consent was given by the patients for their information to be stored in the hospital database and used for research purposes, aligned with the analysis described herein. The current retrospective analysis described herein did not require additional review by said ethics committee due to prior approval of future biomedical analyses when EGIR-RISC study was initiated.

### Research protocol

Electrical bioimpedance (to measure fat-free mass), routine clinical chemistry, OGTT, and HI clamp were performed as described [38]. Insulin sensitivity was expressed as M_FFM_, in units of µmol per min per kg of fat-free mass. Plasma free fatty acids (FFA) were measured in the fasting state and at timed intervals during the clamp; the values during the last 40 min of the clamp were averaged to express insulin inhibition of circulating FFA.

### Metabolomic analysis

Biochemical profiling was performed using multiple platform (UHPLC and GC) mass spectrometry technology, as described [18], [19], [39]. Briefly, a broad array of small molecule metabolites, irrespective of class (*e.g.*, amino acids, lipids, carbohydrates), was examined to measure biochemical changes within plasma samples collected after an overnight (10–12 hours) fast. The non-targeted process used single sample extraction followed by protein precipitation to recover a diverse range of molecules (*e.g.*, polar, hydrophobic).

### Metabolite identification

Metabolites were identified by automated comparison and spectra fitting to a chemical standard library of experimentally derived spectra as previously described [18], [19], [39]. Identification of known chemical entities was based on comparison with library entries of purified authentic chemical standards. 485 biochemicals were identified in this global biochemical profiling analysis, with 350 biochemicals measured in >50% of the entire data set. The latter grouping of 350 biochemicals was used in all of the statistical analyses.

### Sample preparation

Upon receipt of fasted, baseline plasma samples from HI clamps, aliquots were prepared and immediately frozen at −80°C until time of analysis. At time of analysis, samples were thawed on ice and 100 µl was extracted using an automated MicroLab STAR® system (Hamilton Company, Salt Lake City, UT). The samples were extracted using a single extraction with 400 µl of methanol, containing the recovery standards: tridecanoic acid, fluorophenylglycine, chlorophenylalanine and d6-cholesterol. The solvent extraction step was performed by shaking for two minutes using a Geno/Grinder 2000 (Glen Mills Inc., Clifton, NJ). After extraction, the sample was centrifuged and supernatant removed using the MicroLab STAR® robotics system. The extract supernatant was split into four equal aliquots: two for UHPLC/MS, one for GC/MS and one reserve aliquot. Aliquots were placed on a TurboVap® (Zymark) to remove solvent, and dried under vacuum overnight. Samples were maintained at 4°C throughout the extraction process. For UHPLC/MS analysis, extract aliquots were reconstituted in either 0.1% formic acid for positive ion UHPLC/MS, or 6.5 mM ammonium bicarbonate pH 8.0 for negative ion UHPLC/MS. For GC/MS analysis, aliquots were derivatized using equal parts N,O-bistrimethylsilyl-trifluoroacetamide and a solvent mixture of acetonitrile:dichloromethane:cyclohexane (5∶4∶1) with 5% triethylamine at 60°C for 1 hour. The derivatization mixture also contained a series of alkyl benzenes for use as retention time markers.

### GC/MS and UHPLC/MS/MS analysis

UHPLC/MS was carried out using a Waters Acquity UHPLC (Waters Corporation, Milford, MA) coupled to an LTQ mass spectrometer (Thermo Fisher Scientific Inc., Waltham, MA) equipped with an electrospray ionization source. Two separate UHPLC/MS injections were performed on each sample: one optimized for positive ions and one for negative ions. The positive ion analyses were performed first, followed by negative ion analyses. The mobile phase for positive ion analysis consisted of 0.1% formic acid in H_2_O (solvent A) and 0.1% formic acid in methanol (solvent B), while the mobile phase for negative ion analysis consisted of 6.5 mM ammonium bicarbonate, pH 8.0 (solvent A) and 6.5 mM ammonium bicarbonate in 95% methanol (solvent B). The acidic extracts were monitored for positive ions and the basic extracts were monitored for negative ions in independent injections using separate acid/base dedicated 2.1×100 mm Waters BEH C18 1.7 µm particle columns heated to 40°C. The extracts were loaded via a Waters Acquity autosampler and gradient eluted (0% B to 98% B, with an 11 minute runtime) directly into the mass spectrometer at a flow rate of 350 µl/min. The LTQ alternated between full scan mass spectra (99–1000 m/z) and data dependent MS/MS scans, which used dynamic exclusion.

The derivatized samples for GC/MS were analyzed on a Thermo-Finnigan Trace DSQ fast-scanning single-quadrupole MS operated at unit mass resolving power. The GC column was 20 m×0.18 mm with 0.18 µm film phase consisting of 5% phenyldimethyl silicone. The temperature program started with an initial oven temperature of 60°C and was ramped to 340°C, with helium as the carrier gas. The MS was operated using electron impact ionization with a 50–750 amu scan range and was tuned and calibrated daily for mass resolution and mass accuracy.

### Data normalization

Samples were analyzed over the course of two weeks. Each run day was balanced for age, BMI, gender, OGTT, and insulin-mediated total glucose disposal, M_FFM_). Within each day run, samples were completely randomized to avoid group block effects. The raw area counts for each metabolite in each sample were normalized to correct for variation resulting from instrument inter-day tuning differences. For each metabolite, the raw area counts were divided by its median value for each run-day, therefore setting the medians equal to 1 for each day's run. This correctly preserves all variation between samples, yet allows metabolites of widely different raw peak areas to be compared directly on a similar graphical scale. Missing values were assumed to result from areas falling below limits of detection. For each metabolite, missing values were imputed with its observed minimum after the normalization step.

### Data extraction and quality assurance

The data extraction of raw mass spectra data files yielded information that was loaded into a relational database and manipulated without resorting to BLOB manipulation. Once in the database the information was examined and appropriate QC limits were imposed. Peaks were identified using Metabolon's proprietary peak integration software, and component parts were stored in a separate and specifically designed complex data structure.

The median relative standard deviation (MRSD), a quality assurance metric of quantification and measure of instrument variability, was determined to be 8% for a panel of 30 internal standards. Overall process variability (*i.e.*, extraction, recovery, resuspension, and instrument performance) for endogenous biochemicals within technical replicate plasma samples was calculated to be 15% MRSD. These SD values reflected acceptable levels of variability for overall process and instrumentation of the analytical platform.

A variety of data curation procedures were carried out to ensure that a high quality data set was made available for statistical analysis and data interpretation. The QC and curation processes were designed to ensure accurate and consistent identification of true chemical entities, and to remove those representing system artifacts, mis-assignments, and background noise. Metabolon data analysts use proprietary visualization and interpretation software to confirm the consistency of peak identification among the various samples. Library matches for each compound were checked for each sample and corrected if necessary. In addition to rigorous identification, the quality of the automated Metabolyzer integration (basis of quantitation) was verified for each biochemical.

For QA/QC purposes a number of additional samples were included with each day's analysis. Briefly, a selection of internal standards was added to every sample, immediately prior to injection into the instrument. These compounds were carefully chosen in order to not interfere with measurement of endogenous compounds. These QC samples were primarily used to evaluate process control for each study. Additionally, a small aliquot of each experimental sample was pooled together to serve as a technical replicate for duration of the run. This technical replicate sample was injected throughout the platform run day and across all run days, allowing variability in quantitation of all consistently detected biochemicals in the experimental samples to be monitored. With this monitoring, a metric on overall process variability was assigned for the platform's performance based on quantitation of metabolites in actual experimental samples (see results section).

### Statistical Analysis

Data are given as median and [interquartile range]. Classification and Regression Trees (CART), Random Forest (RF) [40], multiple linear regression, correlation, and logistic regression analyses were carried out on untransformed data, whereas log-transformed data were used for *t*-testing. When data from NGT, IGT, or IFG categories were used in comparisons for classification by RF, the number of in-bag samples was set to 50% of smallest sub-group to account for unbalanced samples sizes. For platform screening data and targeted analytical data, we used 50,000 and 1,000 trees, respectively. Random forest analysis was performed using the R-package “randomForest” [41]. Partition analysis (JMP) was employed to find the metabolite value that best separated the M_FFM_ value into two groups. Multiple logistic regression tested the independent association of metabolites with lower tertile of insulin resistance; results are given as the odds ratio and 95% confidence interval (C.I.). Statistical analyses were performed using JMP (JMP, Version 8. SAS Institute Inc., Cary, NC, 1989–2009), and “R” (http://cran.r-project.org/).

### Targeted analytical methods

For absolute quantitation, metabolites were analyzed by isotope dilution UHPLC-MS-MS (except for palmitoleic acid, palmitoyl-lyso-PC, and oleoyl-lyso-PC). 50 µl of EDTA plasma samples were spiked with internal standard solution and subsequently subjected to protein precipitation by mixing with 250 µl of methanol. Following centrifugation, aliquots of clear supernatant were injected onto an UHPLC-MS-MS system, consisting of a Thermo TSQ Quantum Ultra Mass Spectrometer and a Waters Acquity UHPLC system equipped with a column manager module and three different columns. Each sample was analyzed using three different chromatographic systems to cover the various analytes.

α-Hydroxybutyric acid (α-HB), β-hydroxybutyric acid and 3-methyl-2-oxo-butyric acid were eluted with a 0.01% formic acid in water/acetonitrile-methanol (1∶1) gradient on a Waters, Acquity BEH C_18_ column (100 mm×2.1 mm, 1.7 µm) at a mobile phase flow rate of 0.4 ml/min at 40°C. Ionization was achieved by negative HESI mode. Creatine, octanoyl carnitine, decanoyl carnitine, glutamic acid, glycine, serine, threonine, palmitoyl-lyso-PC, oleoyl-lyso-PC and linoleoyl-lyso-PC were eluted with a 0.01% formic acid in water/acetonitrile-water-ammonium formate (700∶300∶2.7) gradient on a Thermo, BioBasic SCX column (50 mm×2.1 mm, 5 µm) at a mobile phase flow rate of 0.5 ml/min at 40°C. Ionization was achieved by positive HESI mode. Palmitic acid, palmitoleic acid, margaric acid, stearic acid, oleic acid, and linoleic acid, were eluted isocratically with 15% 5 mM ammonium bicarbonate in water and 85% acetonitrile-methanol (1∶1) on a Waters, Acquity BEH C_18_ column (100 mm×2.1 mm, 1.7 µm) at a mobile phase flow rate of 0.4 ml/min at 40°C. Ionization was achieved by negative HESI mode. Quantitation was performed based on the area ratios of analyte and internal standard peaks using a weighted linear least squares regression analysis generated from fortified calibration standards in an artificial matrix, prepared immediately prior to each run. The following corresponding stable labeled compounds were used as internal standards: α-HB-D_3_, β-HB-D_3_, 3-methyl-2-oxobutyric acid-D_7_, palmitic acid-^13^C_16_, margaric acid-D_3_, oleic acid-^13^C_18_, stearic acid-D_3_, linoleic acid-^13^C_18_, linolenic acid-^13^C_18_, (used for palmitoleic acid), creatine-D_3_, octanoyl carnitine-D_3_, decanoyl carnitine-D_3_, glutamic acid-D_5_, glycine-^13^C_2_-^15^N, serine-D3, threonine-^13^C_4_-^15^N, tryptophan-D_5_, linoleoyl-lyso-PC-D_9_ (also used for palmitoyl-lyso-PC and oleoyl-lyso-PC).

### Quantitative determination of α-HB

For extraction, 0.0500 mL of human EDTA plasma was spiked with 0.0200 mL α-HB-D_3_ internal standard solution (30.0 µg/mL) and subjected to protein precipitation by vigorously mixing with 0.250 mL of methanol. Following centrifugation, the supernatant was removed and 2.00 µL were injected onto a Waters Acquity/Thermo Quantum Ultra LC-MS-MS system. Calibration range included 0.500 to 20.0 µg/mL α-HB. Calibration standard samples were prepared in 2% BSA or water. Chromatographic conditions included the following: Waters, Acquity C 18 BEH column, 1.7 micron 2.1×100 mm; mobile phase A: 0.01% formic acid in water; mobile phase B: acetonitrile-methanol (1∶1); flow rate: 0.400 mL/min; gradient: initial 99% phase A, 1.0 min 60% phase A, linear, 1.4 min 60% phase A, 1.5 min 99% phase A; and linear α-HB retention time was 1.22 min. Mass spectrometer settings included selective reaction monitoring, negative ionization mode; HESI source; Spray voltage: −2500 V; vaporizer temperature: 300°C, Capillary temperature: 350°C; sheath/auxillary/sweep gas: N_2_; collision gas: Ar, 0.5 mTorr; monitored transitions: α-HB: m/z 103.1->57.1, α-HB-D_3_: m/z 106.1->59.1, collision energy: 13 V, each.

## Supporting Information

Appendix S1 List of EGIR-RISC Investigators and Centers.
(0.03 MB DOC)
