# Supplementary material for: α-Hydroxybutyrate Is an Early Biomarker of Insulin Resistance and Glucose Intolerance in a Nondiabetic Population
Source: PLoS One. 2010 May 28;5(5):e10883. doi: 10.1371/journal.pone.0010883 (PMC2878333; doi:10.1371/journal.pone.0010883)
Supplement: Appendix S1 — List of EGIR-RISC Investigators and Centers. (0.03 MB DOC) [file pone.0010883.s001.doc]

# APPENDIX – Gall *et al*. α-hydroxybutyrate is an early biomarker of insulin resistance and glucose intolerance in a nondiabetic population

**Contact details: EGIR-RISC participating centers and investigators**

**Pisa, Italy - Prof Ele Ferrannini (EGIR-RISC Project Coordinator)**

Department of Internal Medicine, University of Pisa, Via Roma 67, 56100 Pisa, Italy
Tel +39 050 552465 (Secretary to Prof. Ferrannini +39 050 553510)
Fax +39 050 553235 / +39 050 552089
email: [ferranni@ifc.cnr.it]](https://mail.metabolon.com/exchange/wgall/Inbox/RE: AHB manuscript-22.EML/?cmd=editrecipient&Index=-1)

**Dr Michaela Kozakova**

Department of Internal Medicine, University of Pisa, Via Roma 67, 56100 Pisa, Italy

**Dr Amalia Gastaldelli**

Stable Isotope Laboratory, Institute of Clinical Physiology, CNR, Via Moruzzi 1, 56100 Pisa, Italy

**London, UK - Dr Simon Coppack**

Academic Medical Unit, The Royal London Hospital, Whitechapel, London E1 1BB, UK

**Villejuif, France - Dr Beverley Balkau**

INSERM U U780-IFR69, 16 Avenue PV Couturier, F-94807 Villejuif, France

**Amsterdam, The Netherlands - Prof Jacqueline Dekker**

EMGO Institute, Vrije Universiteit Amsterdam, Van der Boechorstrstraat 7, 1081 BT Amsterdam, The Netherlands

**Newcastle-upon-Tyne, UK - Prof Mark Walker**

Department of Medicine, The Medical School, University of Newcastle upon Tyne, Framlington Place, NE2 41A1, UK

**Padova, Italy - Dr Andrea Mari**

ISIB-CNR, Corso Stati Uniti 4, I-35127 Padova, Italy

**Lyon, France - Prof Martine Laville**

Pavillon X, Hopital E Herriot, Place d'Arsonval, F-69003 Lyon, France

**Odense, Denmark - Prof Henning Beck-Nielsen**

Odense University Hospital, Department of Endocrinology M, Sdr Boulevard 29, DK-5000 Odense, Denmark

**Dublin, Ireland - Prof John Nolan**

Department of Endocrinology, St James' Hospital, James' Street, Dublin 8, Ireland

**Perugia, Italy - Prof Geremia Bolli**

DiMI, University of Perugia, Via E Dal Pozzo, I-06126 Perugia, Italy

**Geneva, Switzerland - Prof Alain Golay**

Division of Therapeutical Teaching for Chronic Diseases, University Hospital Geneva, CH-1211 Geneva 14, Switzerland

**Frankfurt, Germany - Dr Thomas Konrad**

Clinic of Pediatrics I, Johnann Wolfgang Goethe Universitat am Main, Theodor Stern-Kai 7, D-60590 Frankfurt, Germany

**Malmö, Sweden - Dr Peter Nilsson and Dr Olle Melander**

Department of Medicine, University Hospital, S-20502 Malmö, Sweden

**Rome, Italy - Dr Geltrude Mingrone**

Istituto di Medicina Interna e Geriatria, Policlinico A Gemelli, Largo A Gemelli 8, I-00168 Rome, Italy

**Glasgow, UK - Dr Colin Perry**

Department of Medicine and Therapeutics, Western Infirmary, Dumbarton Road, Glasgow G11 6NT, UK

**Dr John Petrie**

Ninewells Hospital and Medical School, Dundee DD1 9SY, Scotland UK

**Vienna, Austria - Dr Christian Anderwald and Prof Anton Luger**

Department of Internal Medicine 3, Endocrinology and Metabolism, General Hospital Vienna, Wahringergurtel 18-20, A-1090 Vienna, Austria

**Madrid, Spain - Dr Rafael Gabriel**

Unidad de Investigacion, Hospital Universitario La Paz, Paseo de la Castellana 261, E-28046 Madrid, Spain

**Athens, Greece - Dr Asimina Mitrakou**

National and Kapodistrian University of Athens, Christou Lada 6, 10561 Athens, Greece

**Milano, Italy - Dr Piermarco Piatti**

Unita di Malattie Metaboliche Medicina 1, Istituto Scientifico San Raffaele, via Olgettina 60, I-201 32 Milano, Italy

**Belgrade, Serbia and Montenegro - Prof Nebojsa Lalic**

Institute for Endocrinology, Diabetes Centre, Dr Subotica 13, 11000 Belgrade, Serbia and Montenegro

**Kuopio, Finland - Prof Marku Laakso**

Department of Medicine, Kuopio University Hospital, FIN-70210 Kuopio, Finland
